# nf-core/viralmetagenome: A novel pipeline for untargeted viral genome reconstruction

**DOI:** 10.1093/bioinformatics/btag187

**Published:** 2026-04-29

**Authors:** Joon Klaps, Philippe Lemey, Magda Bletsa, nf-core community, Liana Eleni Kafetzopoulou

**Affiliations:** Rega Institute for Medical Research, Department of Microbiology, Immunology and Transplantation, KU Leuven, Leuven, Belgium; Rega Institute for Medical Research, Department of Microbiology, Immunology and Transplantation, KU Leuven, Leuven, Belgium; Rega Institute for Medical Research, Department of Microbiology, Immunology and Transplantation, KU Leuven, Leuven, Belgium; Rega Institute for Medical Research, Department of Microbiology, Immunology and Transplantation, KU Leuven, Leuven, Belgium; Leiden University Center of Infectious Diseases (LUCID), Leiden University Medical Center, Leiden, 2333 ZA, the Netherlands

## Abstract

**Motivation:**

Reconstructing eukaryotic viral genomes from metagenomic data is challenging due to their extensive diversity and potential genome segmentation. Current approaches often rely on labor-intensive manual curation for reference selection and scaffolding, limiting scalability for large studies or rapid outbreak response. We address the critical need for an automated, scalable pipeline for efficient viral metagenomic analysis without manual intervention.

**Results:**

We present nf-core/viralmetagenome, a comprehensive Nextflow pipeline for the untargeted reconstruction and variant analysis of eukaryotic DNA and RNA viruses from short-read metagenomic or hybridisation capture enriched samples. The pipeline automates the entire process from read preprocessing to consensus generation, integrating multiple *de novo* assemblers, automated reference selection, and iterative consensus refinement. It features robust quality control, extensive documentation, and seamless portability via Docker and Singularity. We validated the pipeline on diverse simulated and real datasets, demonstrating its ability to recover high-quality genomes from complex metagenomic samples and resolve co-infections, making it a powerful tool for viral surveillance.

**Availability:**

nf-core/viralmetagenome is freely available at https://github.com/nf-core/viralmetagenome with comprehensive documentation at https://nf-co.re/viralmetagenome. Archival code repository snapshots are published at zenodo with doi: https://doi.org/10.5281/zenodo.17524074.

## 1. Introduction

Reconstructing viral genomes from metagenomic sequencing data presents considerable computational challenges, particularly for viruses exhibiting extensive genetic diversity (Baaijens et al. 2017, Meleshko et al. 2021). This challenge is amplified in segmented viruses such as influenza, rotavirus, and bunyaviruses, where individual segments can evolve under distinct selective pressures and can undergo reassortment. While some pipelines target specific viruses and their subtypes (Shepard et al. 2016), accurate genome reconstruction from samples with unknown viral strains typically requires time-consuming manual curation of contigs and reference matching (de Vries et al. 2021). This makes large-scale metagenome studies or rapid outbreak responses impractical.

To address these limitations, we developed nf-core/viralmetagenome, a comprehensive pipeline specifically designed for untargeted viral genome reconstruction using short read sequences. The pipeline is developed using Nextflow (Di Tommaso et al. 2017) within the nf-core framework (Langer et al. 2025), ensuring reproducibility through containerization with Docker (Merkel 2014) and Singularity (Kurtzer et al. 2017), as well as portability across computational platforms such as local desktops, high-performance clusters and cloud environments.

## 2. Pipeline description

**Figure 1:**
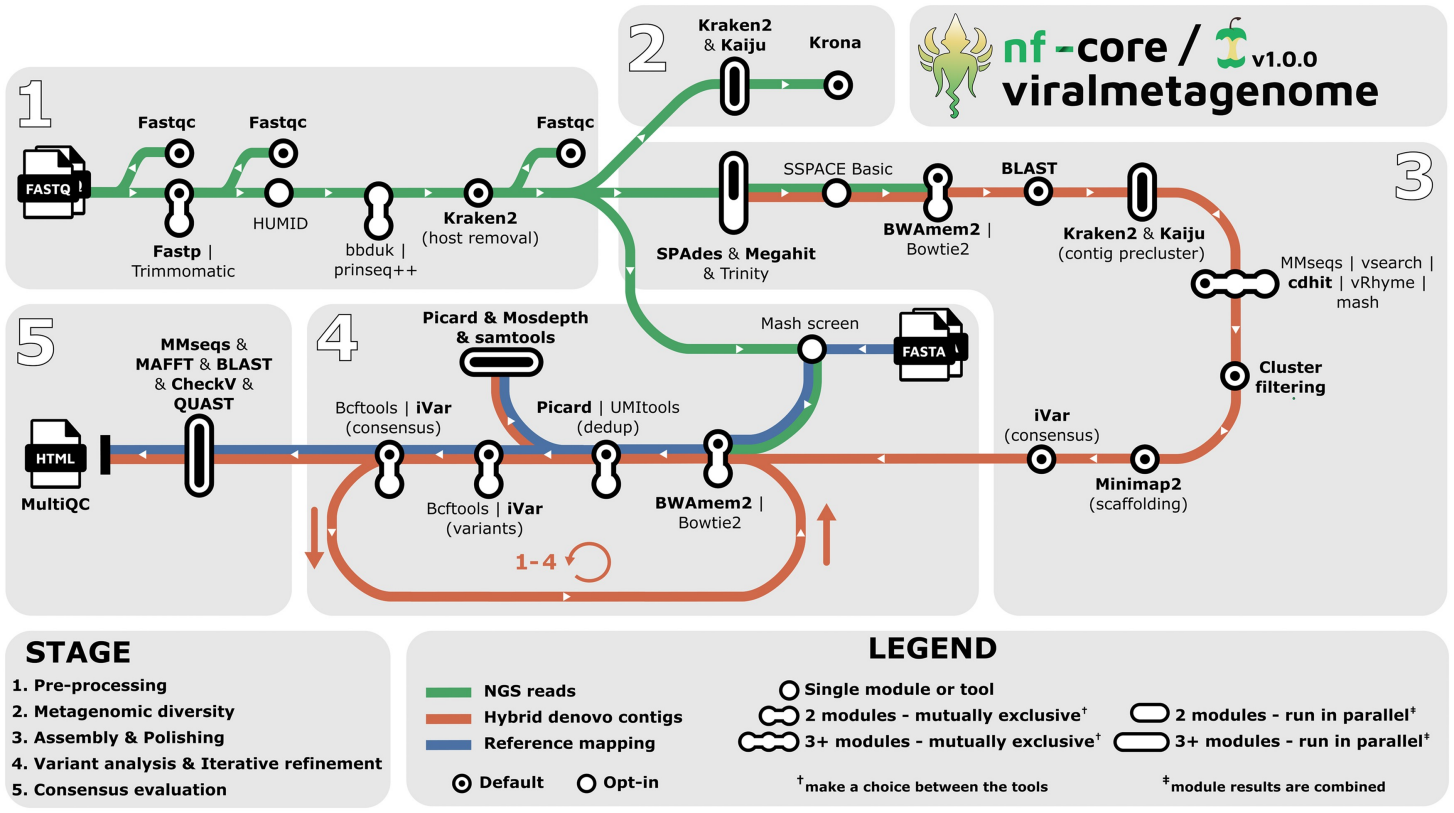
Overview of the nf-core/viralmetagenome pipeline for untargeted viral genome reconstruction. Default options are highlighted in bold. nf-core/viralmetagenome processes short-read data through pre-processing, metagenomic diversity assessment, *de novo* assembly with multiple assemblers, scaffolding with automated reference identification and taxonomy-guided contig clustering, and iterative consensus refinement. Quality-control metrics, assembly statistics, and coverage data are summarised in MultiQC reports and overview tables for downstream analysis.

nf-core/viralmetagenome implements a workflow for *de novo* assembly, reference matching, and iterative consensus refinement to reconstruct viral genomes without prior target knowledge. The pipeline comprises five stages: read preprocessing, metagenomic diversity assessment, contig assembly and scaffolding, iterative consensus refinement with variant analysis, and quality control (Fig. 1). Unless otherwise specified, the pipeline offers multiple options to accommodate established user workflows and preferences. Detailed tool descriptions are provided in Table 1, available as supplementary data at *Bioinformatics* online.

### 2.1. Read preprocessing

Input reads are provided via sample sheets containing sample names and paths to short-read FASTQ files. These are preprocessed using FastQC and trimmed for adapters using fastp (Chen et al. 2018) (default) or Trimmomatic (Bolger et al. 2014). Fastp offers automated adapter detection and faster processing (Chen et al. 2018). UMI deduplication is implemented using HUMID (GitHub repository: https://github.com/jfjlaros/HUMID) for raw reads, or UMI-tools after mapping (Smith et al. 2017). Multiple sequencing runs can be merged after trimming by specifying merge group identifiers in the sample sheet. Optional filtering with bbduk (Bushnell 2014) or PRINSEQ++ (Cantu *et al.* 2019) removes low-complexity sequences, while host reads are removed with Kraken2 (Wood et al. 2019).

### 2.2. Metagenomic diversity assessment

Preprocessed reads are taxonomically classified with Kaiju (Menzel et al. 2016) and Kraken2 (Wood et al. 2019) to maximise sensitivity across diverse viral families. Results from both classifiers are visualised using Krona (Ondov et al. 2011).

### 2.3. *De novo* assembly and clustering

The assembly workflow employs a multi-assembler approach where contigs generated by all tools are pooled and processed jointly. *De novo* assembly uses SPAdes (Meleshko et al. 2021) (RNAviral mode), MEGAHIT (Li et al. 2016), and Trinity (Grabherr et al. 2011), capitalizing on their distinct algorithmic strengths to maximise genome recovery across diverse viral families and read depths. Subsequently, a BLASTn (Altschul et al. 1990) search against the Reference Viral Database (RVDB) (Chin et al. 2025) identifies candidate reference genomes for each contig. All contigs and their identified reference genomes are subjected to clustering. This strategy enables contig scaffolding against the cluster centroid whether a database reference genome or a *de novo* contig, thereby combining outputs from multiple assemblers into unified scaffolds.

Clustering is performed in two sequential stages. First, taxonomic pre-clustering groups contigs based on taxonomic classification using Kraken2 (Wood et al. 2019) and Kaiju (Menzel et al. 2016), with optional filtering to focus on specific taxonomic clades for more targeted analyses. Second, nucleotide similarity clustering within taxonomic groups is performed using CD-HIT-EST (Li and Godzik 2006) (default), VSEARCH (Rognes et al. 2016), MMseqs2 (Steinegger and Söding 2017), vRhyme (Kieft et al. 2022), or Mash (Ondov et al. 2019). All tools are valid options, though performance may vary by dataset (Steinegger and Söding 2017, Zielezinski et al. 2025).

Optionally, following *de novo* assembly and extension, reads can be mapped to all contigs using BWA-MEM2 (Vasimuddin et al. 2019) (default), or Bowtie2 (Langmead et al. 2019). Clusters are filtered based on the total percentage of reads mapping to all contigs within a cluster, removing assembly artefacts. For the final scaffolding step, all cluster members are mapped to the cluster representative or centroid using Minimap2 (Li 2018), followed by consensus calling with iVar (Grubaugh et al. 2019) to generate reference-assisted scaffolds.

### 2.4. Consensus generation and variant calling

The consensus module supports reference-guided analysis and scaffold refinement using user-defined reference genomes. Users can provide one or multiple references; when multiple are provided, Mash (Ondov et al. 2019) selects the most similar one. The selected reference then undergoes a single refinement round to generate the consensus.

In contrast, scaffold refinement employs an iterative approach, performing up to 4 cycles (default 2) to polish the consensus sequence. Both processes map reads using BWA-MEM2 (Vasimuddin et al. 2019) (default), or Bowtie2 (Langmead et al. 2019), followed by variant calling with BCFtools (Danecek et al. 2021) or iVar (Grubaugh et al. 2019) (default). Benchmarking (Bassano et al. 2022) indicated that BCFtools outperforms iVar in precision and recall, while iVar identified more low-frequency variants. Users can prioritise sensitivity or specificity when selecting the variant caller. Final variant calling is performed on the polished consensus to characterise single nucleotide variants.

## 3. Consensus quality control

Quality control employs CheckV (Nayfach et al. 2021) to assess completeness, BLASTn (Altschul et al. 1990) for reference similarity, and MMseqs2 (Steinegger and Söding 2017) against the annotated database Virosaurus (Gleizes et al. 2020). These analyses enable species identification, genomic segment classification, host prediction, and retrieval of additional metadata embedded within the reference databases.

The consensus refinement is evaluated through sequence alignment with MAFFT (Katoh et al. 2002), which compares final consensus genomes against *de novo* contigs, intermediate consensus sequences from iterative cycles, and the scaffolding reference. All tool metrics are compiled into an interactive MultiQC report (Ewels et al. 2016). Additionally, key metrics are extracted from the MultiQC report and compiled into overview tables for downstream analysis.

## 4. Applications

We validated the pipeline’s performance using a diverse set of real metagenomic samples containing human and plant pathogens. nf-core/viralmetagenome successfully reconstructed high-quality or near-complete consensus genomes across various viral families, including segmented viruses (Lassa virus, Orthonairovirus, Tomato spotted wilt tospovirus) and non-segmented viruses (SARS-CoV-2, West Nile virus, Potato virus Y, Youcai mosaic virus, and Monkeypox virus; Supplementary methods S1, available as supplementary data at *Bioinformatics* online). The pipeline proved efficient, processing 28 samples in 412 CPU hours with a maximum memory footprint of 79GB on a high-performance cluster. To facilitate local deployment, we implemented a -profile local option, which utilizes a smaller viral RefSeq database instead of the full RVDB database for Kaiju, reducing the pipeline’s peak RAM usage to approximately 18GB.

To evaluate the pipeline’s robustness in complex scenarios, we conducted two targeted experiments. First, we assessed its ability to resolve co-infections using a simulated mixture of viral genomes (Supplementary Methods S2, available as supplementary data at *Bioinformatics* online). The pipeline successfully distinguished and reconstructed distinct co-infecting strains, provided the genetic divergence was sufficient (≤ 88.7% average nucleotide identity (ANI); Figure 1, available as supplementary data at *Bioinformatics* online).

**Figure 2:**
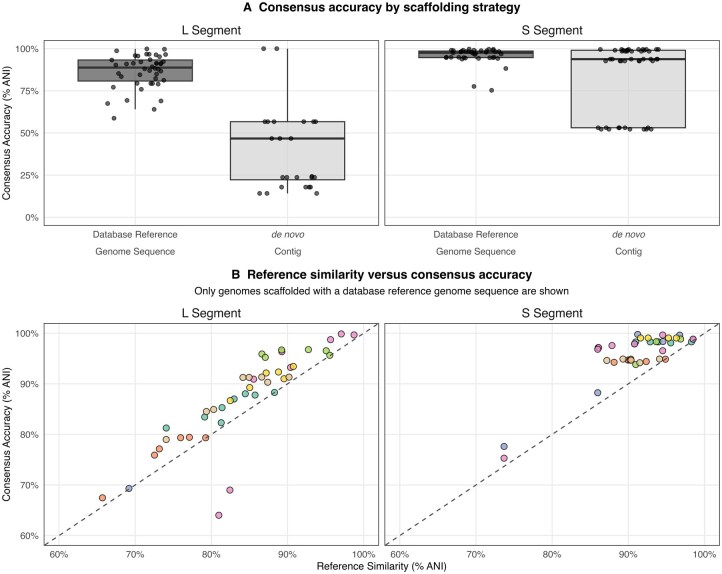
Impact of scaffolding reference similarity on consensus reconstruction accuracy. Consensus accuracy is defined as the ANI (Eq. 1 in Supplementary Methods, available as supplementary data at *Bioinformatics* online) between the test consensus and the baseline consensus (generated using the pipeline’s automated reference selection from the full unclustered-RVDB). Test consensus genomes were scaffolded using either a supplied database reference genome or a *de novo* contig. (A) Boxplots comparing Consensus Accuracy (% ANI) for the L and S segments. The plots contrast the performance of using a database reference genome as the scaffolding backbone (dark grey) against relying solely on the *de novo* contig (light grey). For the low-coverage L segment, using the database reference genome sequences significantly improves accuracy, whereas for the high-coverage S segment, the *de novo* route performs comparably to the database reference genome scaffolding (sequencing depths detailed in Supplementary Figure S4, available as supplementary data at *Bioinformatics* online). (B) Scatter plots illustrate the relationship between reference similarity and consensus accuracy (% ANI) for the L and S segments. Here, reference similarity is defined as the ANI between the supplied database reference genome sequence and the baseline consensus genome. The dashed line represents the line of identity (*y* = *x*), and points are coloured by sample source (Supplementary Figure S2, available as supplementary data at *Bioinformatics* online). Only consensus genomes that used a database reference genome sequence as the scaffold reference are shown in panel B.

The second part of our benchmarking explored the critical role of the scaffolding reference in consensus genome reconstruction. We selected sequencing data from seven Lassa virus-positive human clinical samples with diverse viral loads (Supplementary Methods S3; Figure 2, available as supplementary data at *Bioinformatics* online). In these samples, a significantly higher sequencing depth was observed for the S segment than for the L segment (Figure 4, available as supplementary data at *Bioinformatics* online). To establish a benchmark, a baseline consensus genome was generated for each sample using automated reference selection from the full unclustered-RVDB (U-RVDB) reference pool. We then systematically challenged the scaffolding process by providing reference genomes (19–30 per sample; Table 2, available as supplementary data at *Bioinformatics* online) spanning a range of similarity to the baseline consensus (64–100% ANI; Figure 3, available as supplementary data at *Bioinformatics* online). For each supplied reference, a new consensus genome was generated using it as the scaffolding backbone when clustered with the *de novo* contigs.

Comparing these generated sequences against their respective baseline consensus highlights the utility of reference scaffolding (Fig. 2), particularly in low read depth scenarios when *de novo* assembly was challenging. Here, we defined consensus accuracy as the Average Nucleotide Identity between each test consensus and its baseline (Supplementary Methods S3, available as supplementary data at *Bioinformatics* online). For the high-coverage S segment (Figure 4, available as supplementary data at *Bioinformatics* online), *de novo* assembly was sufficient to reconstruct the full genome, rendering the choice of scaffolding reference largely redundant (Fig. 2A). In contrast, the low-coverage L segment proved more challenging: *de novo* assembly produced shorter, incomplete fragments, demonstrating that the pipeline’s ability to identify and use a closely related database reference genome was essential to bridge gaps and recover a complete segment consensus sequence.

These findings emphasise the need to keep databases like RVDB (Chin et al. 2025) up-to-date. Since nf-core/viralmetagenome is primarily designed for eukaryotic viruses, users interested in bacteriophage analysis are encouraged to explore pipelines designed to target phages such as VIRify (Rangel-Pineros et al. 2022), VIBRANT (Kieft et al. 2020), VirSorter2 (Guo et al. 2021).

## 5. Conclusion

nf-core/viralmetagenome addresses a critical need in viral genomics by providing an automated, scalable solution for untargeted viral genome reconstruction. The pipeline successfully automates the traditionally time-consuming and manual process of viral genome reconstruction from short-read metagenomic data through its integrated workflow of *de novo* contig assembly, automated reference selection, clustering algorithms, and iterative refinement strategies.

Our validation results indicate that the pipeline is suitable for a wide range of eukaryotic viral families, it removes the burden of extensive manual curation, and enables consistent, high-quality genome reconstruction with reproducible results across varied computational settings. As viral surveillance and outbreak response increasingly rely on metagenomic sequencing, automated pipelines like nf-core/viralmetagenome will be essential for the timely identification of pathogen strains. The pipeline represents a significant step forward in making viral genome reconstruction accessible to researchers without requiring extensive bioinformatics expertise, facilitating broader adoption of metagenomic approaches in viral research and public health applications.

## Supplementary Material

btag187_Supplementary_Data

## Data Availability

nf-core/viralmetagenome is freely available at https://github.com/nf-core/viralmetagenome with comprehensive documentation at https://nf-co.re/viralmetagenome. Archival code repository snapshots are published at zenodo with doi: https://doi.org/10.5281/zenodo.17524074.
