# Supplementary material for: nf-core/viralmetagenome: A novel pipeline for untargeted viral genome reconstruction
Source: Bioinformatics. 2026 Apr 29;42(5):btag187. doi: 10.1093/bioinformatics/btag187 (PMC13141149; doi:10.1093/bioinformatics/btag187)
Supplement: btag187_Supplementary_Data [file btag187_supplementary_data.zip › supplementary-methods.pdf]

Supplementary Methods & Tables - nf-core/viralmetagenome: A Novel Pipeline for Untargeted Viral Genome Reconstruction

Joon Klaps[1,*], Philippe Lemey[1], Magda Bletsa[1], nf-core community[2], Liana Eleni Kafetzopoulou[1,3]

[1]Rega Institute for Medical Research, Department of Microbiology, Immunology and Transplantation, KU Leuven, Belgium

[2]A full list of contributors can be found at https://nf-co.re/community.

[3]Leiden University Center of Infectious Diseases (LUCID), Leiden University Medical Center, Albinusdreef 2, 2333 ZA, Leiden, the Netherlands

*Correspondence: joon.klaps@kuleuven.be

April 13, 2026

Data Availability

The nf-core/viralmetagenome pipeline is freely available at `https://github.com/nf-core/viralmetagenome`. Scripts used to generate the results as well as parts of the results are available on GitHub at `https://github.com/Joon-Klaps/nf-core-viralmetagenome-manuscript`.

Supplementary Methods

Throughout this manuscript, sequence similarity is quantified using Average Nucleotide Identity (ANI), defined as:

$$\text{ANI} = \frac{N_{\text{matches}}}{L_{\text{global alignment}}} \tag{1}$$

where $L_{\text{global alignment}}$ is the total length of the pairwise global alignment and N_{matches} is the number of exact matches, counting only identical nucleotide pairs (A–A, C–C, G–G, T–T). Figure 2 (S3) and Supplementary Fig. S1 (S2) use this definition of ANI to evaluate reconstruction accuracy.

S1. Evaluation of nf-core/viralmetagenome on public data

A metagenomic dataset was constructed from publically available data by selecting a random subset of 2,000 records from the NCBI Virus database, targeting the following viruses: *Orthonairovirus haemorrhagiae*, *Mammarenavirus lassaense*, *Zika virus*, *West Nile virus*, *Monkeypox virus*, *Influenza A virus*, *Severe acute respiratory syndrome coronavirus 2*, and *Human respiratory syncytial virus A*. Records were filtered to retain only those with associated SRA data, sequenced on the Illumina platform, and with a LibraryStrategy other than 'Amplicon'. From the resulting pool, 28 samples were randomly selected and downloaded using nf-core/fetchngs. Samples were then

run with nf-core/viralmetagenome using default options with the exception of `-cluster_method 'mmseqs-cluster'` due to sequence size limitations of cdhit, and `-skip_read_classification` to speed up the analysis.

For plant pathogens, two samples were randomly selected from the SRA at every query: *Tobacco mosaic virus*, *Tomato spotted wilt virus*, *Cucumber mosaic virus*, and *Potato virus Y*. These were also downloaded using nf-core/fetchngs and processed with viralmetagenome using the same parameters (`-cluster_method 'mmseqs-cluster'`, `-skip_read_classification`), with the addition of the Virosaurus plant virus database as annotation database (`-annotation_db https://ftp.expasy.org/databases/viralzone/2020_4/virosaurus98_plant-20200330.fas.gz`) and RVDB's v29.0 unclustered database as reference pool (`-reference_pool https://rvdb.dbi.udel.edu/download/U-RVDBv29.0.fasta.gz`).

S2. Sample mixture resolution

To evaluate the pipeline's ability to distinguish viral genomes from the same species in mixed samples, we simulated a set of HIV-1 co-infections. We retrieved a random subset of 2,000 HIV-1 genomes (taxid: 11676) from NCBI Virus and selected MN090277.1 as the constant reference genome. We then calculated the pairwise Average Nucleotide Identity (ANI) between all 2,000 genomes and the constant reference genome (MN090277.1) using MMseqs2 (Steinegger and Söding 2017). Based on these ANI values, we binned the genomes into 0.5% intervals (ranging from 74% to 100%) and selected one representative genome from each bin (n=38, Supplementary Table S3). Synthetic paired-end reads were generated for both the constant reference genome and the representative genome using InSilicoSeq v2.0.1(Gourlé et al. 2019) in 'kde' mode with the 'MiSeq' error model. Mixtures were simulated at a 50:50 abundance ratio with 50x coverage for each genome.

The simulated mixtures were analyzed using viralmetagenome with the parameters `-skip_preprocessing`, `-skip_read_classification`, and with U-RVDBv29.0 as a reference pool. To assess performance, we compared all generated consensus sequences with the two reference genomes used to generate the synthetic read mixture (the constant reference genome MN090277.1 and the representative genome for each bin). We performed global alignments using MAFFT (Katoh et al. 2002) and determined their alignment statistics (number of exact matches, approximate matches, number of mismatches, and alignment length). For each generated consensus, we determined the closest matching reference (i.e. the constant reference genome MN090277.1 or the representative reference genome) based on the highest number of exact matches in the global alignment. We identified three distinct performance zones based on empirical ANI thresholds (Supplementary Fig. S1):

- **Unresolved Zone (>88.7%):** When the genetic similarity between the constant reference sequence MN090277.1 and the representative reference genome in the mixture is relatively high, the pipeline is unable to distinguish which reference genome the consensus sequence corresponds to. This leads to the generation of one single mixed consensus sequence for each interval bin in this high genetic similarity range.
- **Ambiguous Zone (85.8% – 88.7%):** In this intermediate zone, the pipeline successfully resolves the simulated mixture; however, the sequence similarity still permits reads to cross-map between references. This causes the reconstructed consensus sequences to accumulate

mismatches in shared regions, resulting in lower fidelity to the original reference genomes despite initial separation by the assemblers.

- **High-Fidelity Resolution ($<$ 85.8%):** Below this similarity threshold, the reference genomes used to create the simulated mixture are sufficiently divergent to minimize cross-mapping interference, allowing for the accurate reconstruction of distinct, high-quality consensus genomes.

S3. Influence of scaffolding reference

The choice of scaffolding reference can considerably influence the completeness of the reconstructed genome. To quantify this effect, we analysed a subset of 7 Lassa virus samples from our own sequencing efforts (Lassa virus - Illumina Miseq data with UMIs). Samples were randomly selected based on their measured viral load during diagnostic testing, ensuring that the dataset encompassed different viral load categories (Supplementary Fig. S2). We compared consensus genomes generated using a gradient of scaffolding reference sequences ranging from 64% to 100% Average Nucleotide Identity (ANI) relative to the sample's baseline consensus.

To establish this gradient, we first generated a baseline consensus for each sample using the unclustered RVDB (U-RVDBv29.0) as the reference pool. We then retrieved all *Mammarenavirus lassaense* (taxid: `3052310`) sequences from NCBI on the 24th of October 2025 (n = 2804) and calculated their ANI against these baseline consensus genomes using MMseqs2 (Steinegger and Söding 2017). From this pool, we selected one scaffolding reference genome at every 2% ANI interval for each sample (Supplementary Fig. S3). In total, 157 reference sequences were selected across the 7 samples (19–30 references per sample), spanning both L and S segments (Supplementary Table S2). The Lassa virus samples exhibited differential coverage between segments (S segment median 231X, L segment median 68X; Supplementary Fig. S4), allowing us to evaluate scaffolding performance on both complete and fragmented assemblies.

By default, nf-core/viralmetagenome selects the cluster centroid as a scaffolding reference. To assess the reference influence, the pipeline's code needed to be slightly modified to force NCBI genomes as scaffolding reference. This version of the code repository is available in the release `https://github.com/Joon-Klaps/viralmetagenome/releases/tag/v1.0.0-force-external-centroid`.

The pipeline was then run with the following non-default parameters: `-with_umi`, `-umi_deduplicate both`, `-skip_read_classification`, `-skip_precluster`, `-intermediate_consensus_caller ivar`, `-consensus_caller ivar`.

To quantify the impact of the scaffolding reference on the final output, we performed global alignments between each test consensus genome (generated with a forced scaffolding reference) and the corresponding baseline consensus genome (generated with automatic reference selection from U-RVDB) using MAFFT (Katoh et al. 2002) and determined their alignment statistics (number of exact matches, number of mismatches, and alignment length).

Figure 2B shows only consensus genomes for which the supplied database reference genome sequence was included in the cluster and used as scaffold reference. During the clustering step (CD-HIT-EST with a default similarity threshold of 0.85 in this benchmark), supplied database reference genome sequences too divergent from the *de novo* contigs are excluded from the cluster. In such cases, the pipeline proceeds without a database reference genome sequence, producing the same *de novo*-only

output regardless of which distant reference was supplied. Consequently, Figure 2B contains fewer data points than the total number of selected references shown in Supplementary Fig. S3.

We observe an almost linear correlation between reference similarity and consensus accuracy for the L segment when database reference genomes were used for scaffolding (Figure 2B; Supplementary Fig. S4A–B). For the S segment, this pattern is less apparent as *de novo* assemblers could reconstruct near-complete genomes for most samples (Figure 2B; Supplementary Fig. S4C–D). In the L segment, where coverage was lower, nf-core/viralmetagenome was particularly effective by connecting individual contigs to improve completeness, but the accuracy was dependent on the similarity of the scaffolding reference.

Supplementary Figures

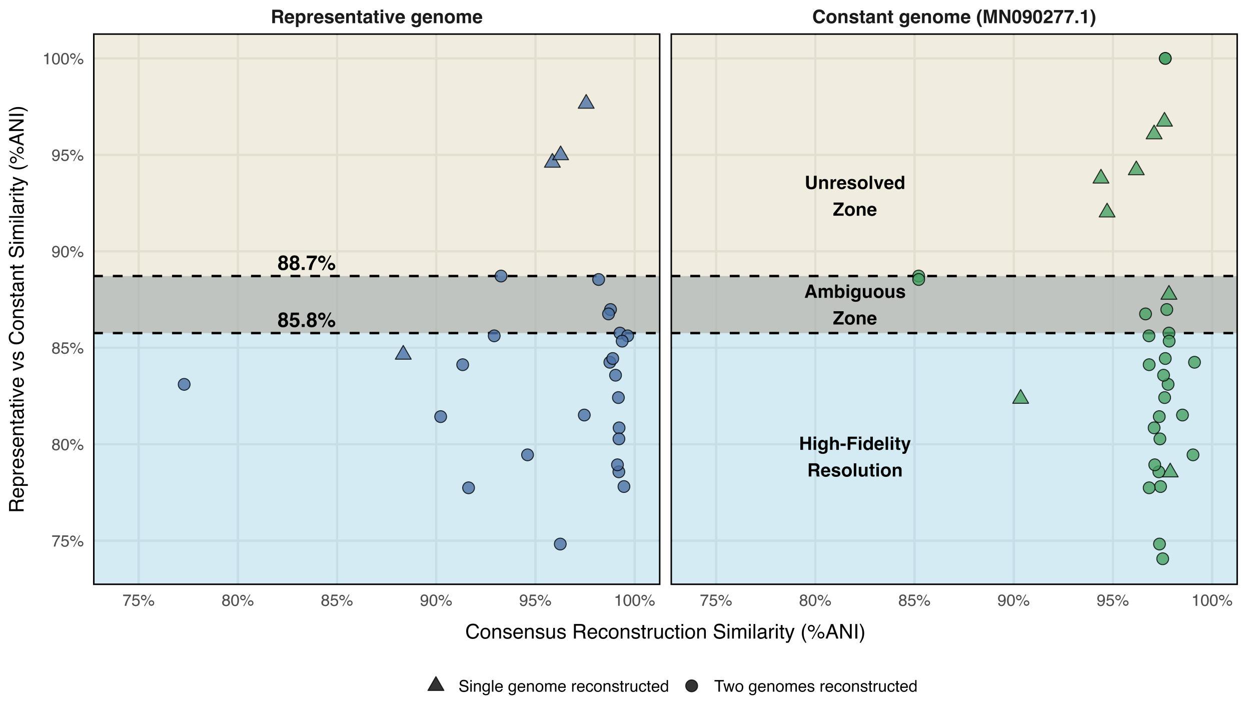

Supplementary Fig. S1: Mixture resolution dynamics across a genetic diversity gradient. This scatter plot characterises nf-core/viralmetagenome's ability to resolve co-infections of varying genetic similarity. The y-axis (Representative vs Constant Similarity) represents the ANI (Eq. 1) between the representative genome and the constant genome MN090277.1. The x-axis shows the ANI between each reconstructed consensus genome and the genome represented in the corresponding panel: the representative genome in the left panel and the constant genome MN090277.1 in the right panel. Blue points in the left panel are consensus genomes whose best match is the representative genome; green points in the right panel are consensus genomes whose best match is the constant genome. Triangles represent a failure in resolving the mixture as only a single genome was reconstructed, circles represent a successful identification of both the constant genome and the representative genome. A perfect mixture resolution yields a blue point near 100% ANI in the representative genome panel and a green point near 100% ANI in the constant genome panel. Three empirically determined zones are highlighted: Unresolved (>88.7% Representative vs Constant Similarity), Ambiguous (85.8–88.7%), and High-Fidelity Resolution (<85.8%).

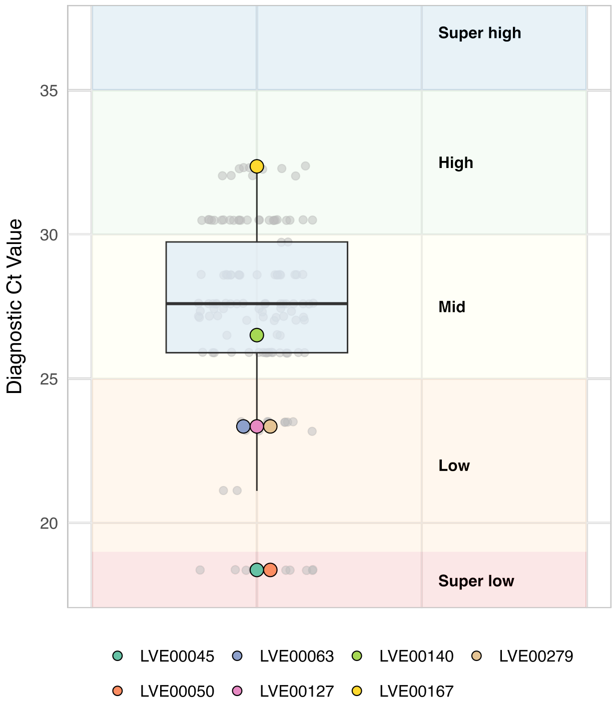

Supplementary Fig. S2: Distribution of diagnostic viral loads. The distribution of diagnostic PCR Ct values for the available Lassa virus samples. Samples are classified into empirical categories (Very low, Low, Mid, High, Very high) based on their Ct values. Up to 3 random samples were selected from each category to ensure a diverse representation of viral loads.

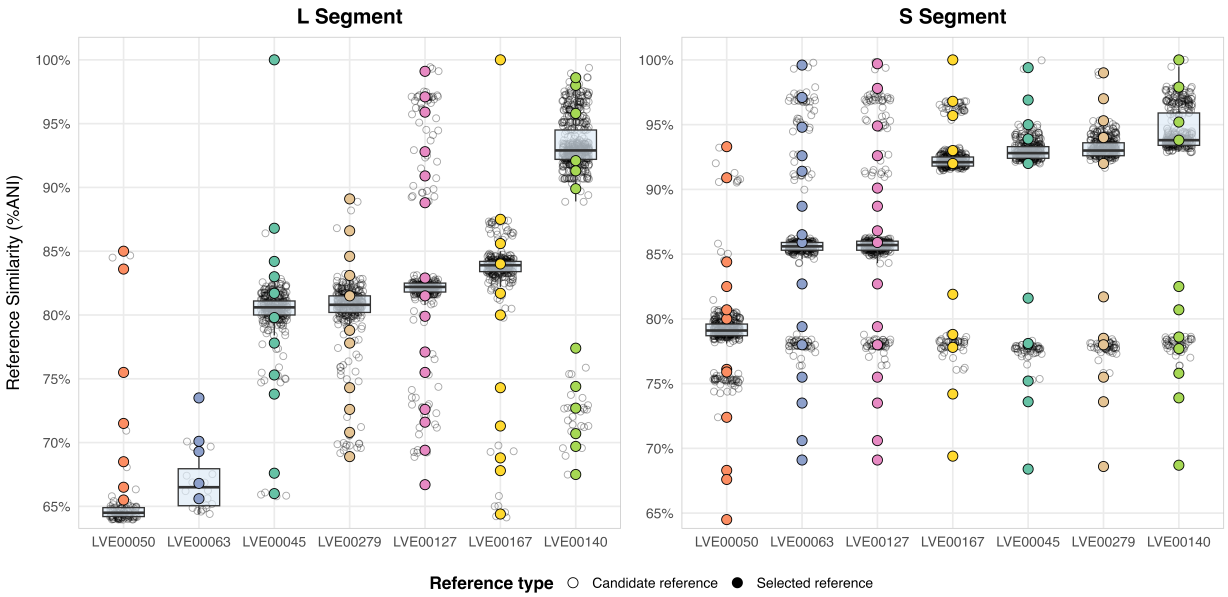

Supplementary Fig. S3: Selection of scaffolding reference genomes across a similarity gradient. To assess how reference divergence affects scaffolding, we compared all available NCBI *Mammarenavirus lassaense* genomes against the baseline consensus (generated with U-RVDB as a reference library supplied with `-reference_pool`) for 7 Lassa virus samples. The y-axis displays the similarity (% ANI) of these candidate references towards the baseline consensus. For the benchmarking experiment, specific references were selected at ∼2% ANI intervals, highlighted in full dots, to establish a gradient of similarity ranging from 64% to 100%.

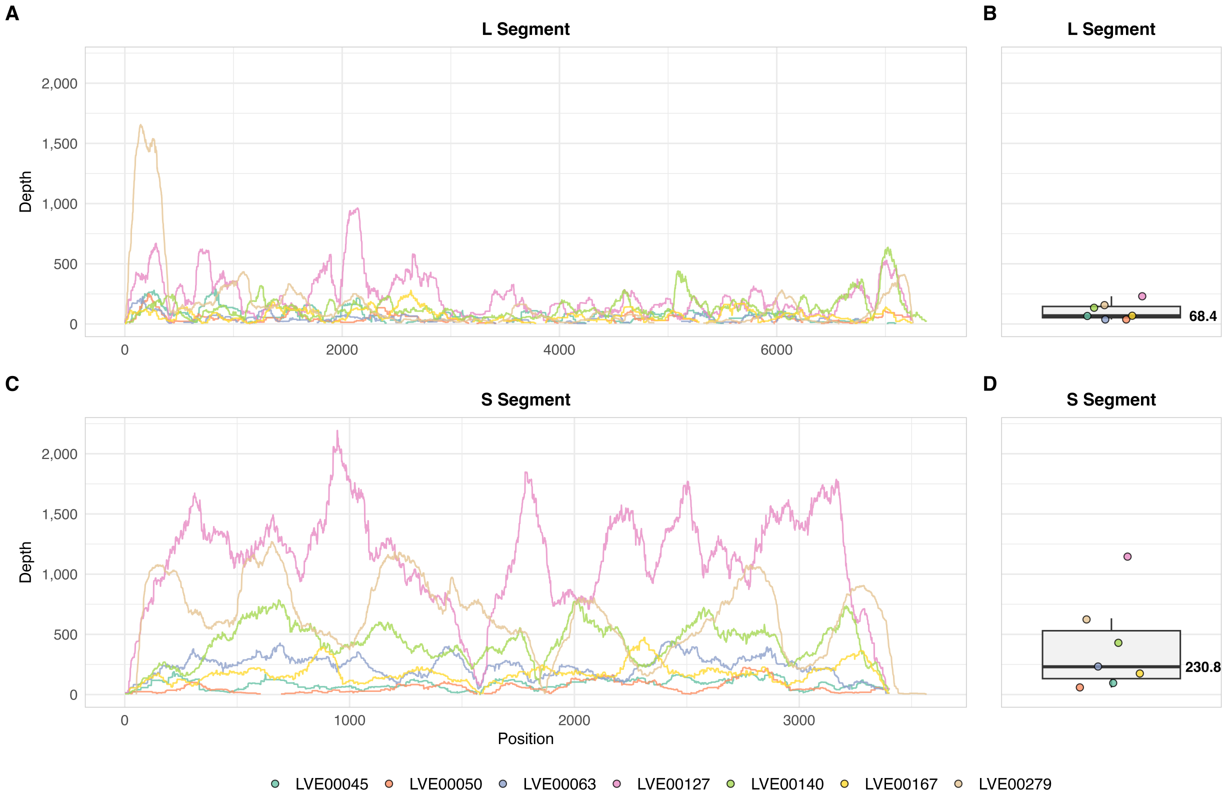

Supplementary Fig. S4: Sequencing coverage across Lassa virus baseline consensus genomes. Consensus genomes used to determine the coverages are the baseline consensus genomes (generated with U-RVDB as a reference library supplied with `-reference_pool`). A,C display per-base coverage depth ($\log_{10}$ scale) along the genome. B,D display mean coverage per consensus. Overall, the S segment has a high read depth (median ~231X), whereas the L segment shows lower coverage (median ~68X) along with multiple areas of zero coverage, presenting a challenge for de novo assembly.

Supplementary Tables

Supplementary Table S1: Overview of computational tools and methods used in the nf-core/viralmetagenome pipeline. The pipeline integrates multiple bioinformatics tools across different stages of viral metagenomic analysis, from preprocessing to consensus calling. Each tool was selected for its specific capabilities and performance characteristics relevant to viral sequence analysis.

Stage	Step	Tool	Explanation	Reference
Pre-processing	Read trimming	fastp	A high-performance preprocessing solution built in C++ that consolidates multiple quality control operations into a single workflow. Fastp delivers enhanced processing speed compared to traditional alternatives, like Trimmomatic, while handling adapter removal, quality assessment, base correction, read and filtering. Fastp also features automated adapter detection capabilities for various Illumina sequencing protocols.	(Chen et al. 2018)
		Trimmomatic	A comprehensive preprocessing solution tailored for Illumina sequencing platforms with specialised paired-end read handling capabilities. Provides multiple pre-processing options, including adapter removal, quality-based trimming using sliding window approaches, and length-based filtering.	(Bolger et al. 2014)
	Complexity filtering	bbduk	A high-performance, multi-threaded tool designed to combine various data-quality-related operations, including trimming, filtering, and masking, into a single pass. It is particularly effective for removing host and other contaminant DNA using k-mer matching strategies, which is crucial for accurate metagenomic analysis. (Website: `https://bbmap.org`)	(Bushnell 2014)
		PRINSEQ++	A C++ implementation that significantly improves upon its predecessor, prinseq-lite.pl, in terms of computational efficiency. It offers extensive quality control features, including filtering by length, GC content, quality scores, N content, and sequence complexity (entropy/DUST score).	(Cantu et al. 2019)

Continued on next page

Table S1 continued from previous page

**Stage**	**Step**	**Tool**	**Explanation**	**Reference**
Metagenomic diversity	Taxonomy classification	Kraken2	An advanced taxonomic classification system with an improved k-mer-based approach through optimised memory usage and enhanced processing speed. Incorporates protein-level search capabilities for improved detection of divergent viral sequences, with a library of pre-built RefSeq indexes on `https://benlangmead.github.io/aws-indexes/k2`	(Wood et al. 2019)
		Kaiju	A protein-level taxonomic classifier that employs exact matching algorithms on translated sequences using the Burrows-Wheeler transform. It can optionally allow amino acid substitutions and tends to have higher sensitivity and precision compared to k-mer-based classifiers (Kraken2). Kaiju is particularly effective for organisms with limited reference representation. A library of pre-built indexes, including RVDB, can be found on `https://bioinformatics-centre.github.io/kaiju/downloads.html`.	(Menzel et al. 2016)
Assembly & polishing	Assembly	SPAdes	A de Bruijn graph-based assembler designed to overcome challenges in complex sequencing data, such as non-uniform coverage (using multisized de Bruijn graphs) and variable insert sizes. The Spades suite contains multiple specialised modes rnaSPAdes, coronaSPAdes, metaSPAdes for extra flexibility.	(Meleshko et al. 2021)
		MEGAHIT	A succinct de Bruijn graph-based assembler specifically designed for large and complex metagenomic datasets. MEGAHIT has demonstrated the ability to generate larger assemblies with longer contig N50 and average contig length. Its relatively low memory requirements make it well-suited for handling large datasets.	(Li et al. 2016)

Continued on next page

Table S1 continued from previous page

Stage	Step	Tool	Explanation	Reference
		Trinity	A powerful modular transcriptome reconstruction tool, capable of fully reconstructing a large fraction of transcripts, including alternative splice isoforms and transcripts from recently duplicated genes, even in the absence of a reference genome. It partitions sequence data into individual de Bruijn graphs, processing each independently to extract full-length isoforms and resolve paralogous genes. Trinity's sensitivity is comparable to methods relying on genome alignments.	(Grabherr et al. 2011)
	Read alignment	BWAmem2	An optimized version of the BWA-MEM algorithm enhanced with Intel-specific acceleration feature, providing approximately a twofold speedup in alignment throughput over BWA-MEM while ensuring identical SAM output. It is highly efficient for aligning short reads to large reference genomes.	(Vasimuddin et al. 2019)
		Bowtie2	A fast and memory-efficient alignment tool for aligning sequencing reads to long reference sequences, such as mammalian genomes. Bowtie2 supports gapped, local, and paired-end alignment modes, combining high speed, sensitivity, and accuracy	(Langmead et al. 2019)
Clustering		CD-HIT-EST	A highly efficient and widely used program for clustering large sets of protein or nucleotide sequences. Implementation constraints limit processing to genomes under 10Mbp with minimum 0.8. The program employs a greedy incremental algorithm.	(Li and Godzik 2006)
		MMSeqs	A powerful software suite for fast and sensitive deep clustering and searching of large protein sequence sets. It is significantly faster than other tools like BLASTclust and can cluster large databases down to low sequence identity thresholds. MMseqs offers a variety of clustering modes for a lot of flexibility.	(Steinegger and Söding 2017)
		VSEARCH	An open-source, versatile alternative for USEARCH. VSEARCH supports processing very large datasets, limited primarily by available memory.	(Rognes et al. 2016)

Continued on next page

Table S1 continued from previous page

Stage	Step	Tool	Explanation	Reference
		Mash	A non-alignment based clustering technique that applies MinHash algorithms for rapid genome and metagenome distance estimation. It compresses large sequences into small, representative sketches, enabling ultra-fast estimations of global mutation distances. Mash creates compact sequence representations enabling ultra-fast similarity assessments and large-scale clustering operations, though accuracy decreases below 95% genome similarity.	(Ondov et al. 2019)
		vRhyme	A machine learning-based binning solution specifically developed for viral genome recovery from metagenomic datasets. VRhyme addresses the unique challenges of viral sequences, such as the lack of universal marker genes. Addresses unique viral sequence characteristics through supervised learning approaches combined with coverage-based analysis across multiple samples. Generates high-quality viral bins with minimal computational overhead, though it may produce empty results when insufficient viral evidence is detected.	(Kieft et al. 2022)
Variant & consensus calling	Variant & consensus calling	iVar	A computational package specifically designed for viral amplicon-based sequencing, integrating functions like consensus and variant calling (including iSNVs and insertions/deletions). It is a key component of best-practice pipelines for reconstructing consensus genomes from viral sequencing data.	(Grubaugh et al. 2019)
		BCFtools	A robust suite of utilities for manipulating variant calls in VCF and BCF formats. Provides extensive functionality for variant calling using multiallelic models, data filtering, file merging, and consensus sequence generation through variant application.	(Danecek et al. 2021)

Supplementary Table S2: Overview of the number of selected references used for each sample to determine the influence of scaffolding reference (Figure 2; Supplementary Fig. S3). References were systematically selected to span a gradient of similarity to each sample's baseline consensus genome, with one reference selected per 2% ANI interval (e.g. 64–66%, 66–68%, . . . , 98–100%).

Sample ID	Total References	L Segment	S Segment
LVE00045	22	13	9
LVE00050	19	7	12
LVE00063	20	11	9
LVE00127	30	16	14
LVE00140	23	13	10
LVE00167	21	11	10
LVE00279	22	12	10

Supplementary Table S3: Overview of the used references for the used sample mixtures (Supplementary Fig. S1). Every 0.5% ANI interval was represented by a single reference (representative reference genome), spanning a gradient of similarity to the constant reference genome (MN090277.1).

Mixture ID	Bin Range	Bin Representative	Constant Genome
HM215250.1_7474	73.5–74.0%	HM215250.1	MN090277.1
MK457885.1_8080	79.5–80.0%	MK457885.1	MN090277.1
PV207457.1_8080	80.0–80.5%	PV207457.1	MN090277.1
MK458218.1_8081	80.5–81.0%	MK458218.1	MN090277.1
MK458244.1_8182	81.0–81.5%	MK458244.1	MN090277.1
MK457977.1_8282	81.5–82.0%	MK457977.1	MN090277.1
MZ766594.1_8282	82.0–82.5%	MZ766594.1	MN090277.1
MT194498.1_8283	82.5–83.0%	MT194498.1	MN090277.1
AF443074.1_8384	83.0–83.5%	AF443074.1	MN090277.1
KC156117.1_8484	83.5–84.0%	KC156117.1	MN090277.1
MN090437.1_8484	84.0–84.5%	MN090437.1	MN090277.1
MW006075.1_8485	84.5–85.0%	MW006075.1	MN090277.1
MN090406.1_8586	85.0–85.5%	MN090406.1	MN090277.1
MN187301.1_8686	85.5–86.0%	MN187301.1	MN090277.1
MN090741.1_8686	86.0–86.5%	MN090741.1	MN090277.1
FJ670529.1_8687	86.5–87.0%	FJ670529.1	MN090277.1
MW063100.1_8788	87.0–87.5%	MW063100.1	MN090277.1
MT929422.1_8888	87.5–88.0%	MT929422.1	MN090277.1
KU168259.1_8888	88.0–88.5%	KU168259.1	MN090277.1
MT191024.1_8889	88.5–89.0%	MT191024.1	MN090277.1
PV738262.1_8990	89.0–89.5%	PV738262.1	MN090277.1
AY835773.1_9090	89.5–90.0%	AY835773.1	MN090277.1
U69588.1_9090	90.0–90.5%	U69588.1	MN090277.1
U69584.1_9091	90.5–91.0%	U69584.1	MN090277.1
MN043582.1_9192	91.0–91.5%	MN043582.1	MN090277.1
MW881692.1_9292	91.5–92.0%	MW881692.1	MN090277.1
K02083.1_9292	92.0–92.5%	K02083.1	MN090277.1
K02013.1_9293	92.5–93.0%	K02013.1	MN090277.1
AY835777.1_9394	93.0–93.5%	AY835777.1	MN090277.1
MN090236.1_9494	93.5–94.0%	MN090236.1	MN090277.1
MN090204.1_9596	95.0–95.5%	MN090204.1	MN090277.1
MN090294.1_9696	95.5–96.0%	MN090294.1	MN090277.1
MN090187.1_9898	98.0–98.5%	MN090187.1	MN090277.1
MN090316.1_9899	98.5–99.0%	MN090316.1	MN090277.1
MN090271.1_99100	99.0–99.5%	MN090271.1	MN090277.1
MN090277.1_100100	99.5–100.0%	MN090277.1	MN090277.1

References

Anthony M Bolger, Marc Lohse, and Bjoern Usadel. Trimmomatic: a flexible trimmer for illumina sequence data. *Bioinformatics*, 30(15):2114–2120, 2014. doi: 10.1093/bioinformatics/btu170.

Brian Bushnell. BBMap: A Fast, Accurate, Splice-Aware Aligner, 2014.

Vito Adrian Cantu, Jeffrey Sadural, and Robert Edwards. PRINSEQ++, a multi-threaded tool for fast and efficient quality control and preprocessing of sequencing datasets. *PeerJ*, 2019. doi: 10.7287/peerj.preprints.27553v1.

Shifu Chen, Yanqing Zhou, Yaru Chen, and Jia Gu. fastp: an ultra-fast all-in-one FASTQ preprocessor. *Bioinformatics*, 34(17):i884–i890, 2018. doi: 10.1093/bioinformatics/bty560.

Petr Danecek, et al. Twelve years of SAMtools and BCFtools. *Gigascience*, 10(2), 2021. doi: 10.1093/gigascience/giab008.

Hadrien Gourlé, Oskar Karlsson-Lindsjö, Juliette Hayer, and Erik Bongcam-Rudloff. Simulating illumina metagenomic data with InSilicoSeq. *Bioinformatics*, 35(3):521–522, 2019. doi: 10.1093/bioinformatics/bty630.

Manfred G Grabherr, et al. Full-length transcriptome assembly from RNA-seq data without a reference genome. *Nat. Biotechnol.*, 29(7):644–652, 2011. doi: 10.1038/nbt.1883.

Nathan D Grubaugh, et al. An amplicon-based sequencing framework for accurately measuring intrahost virus diversity using PrimalSeq and iVar. *Genome Biol.*, 20(1):8, 2019. doi: 10.1186/s13059-018-1618-7.

Kazutaka Katoh, Kazuharu Misawa, Kei-Ichi Kuma, and Takashi Miyata. MAFFT: a novel method for rapid multiple sequence alignment based on fast fourier transform. *Nucleic Acids Res.*, 30 (14):3059–3066, 2002. doi: 10.1093/nar/gkf436.

Kristopher Kieft, et al. vRhyme enables binning of viral genomes from metagenomes. *Nucleic Acids Res.*, 50(14):e83, 2022. doi: 10.1093/nar/gkac341.

Ben Langmead, Christopher Wilks, Valentin Antonescu, and Rone Charles. Scaling read aligners to hundreds of threads on general-purpose processors. *Bioinformatics*, 35(3):421–432, 2019. doi: 10.1093/bioinformatics/bty648.

Dinghua Li, et al. MEGAHIT v1.0: A fast and scalable metagenome assembler driven by advanced methodologies and community practices. *Methods*, 102:3–11, 2016. doi: 10.1016/j.ymeth.2016.02.020.

Weizhong Li and Adam Godzik. Cd-hit: a fast program for clustering and comparing large sets of protein or nucleotide sequences. *Bioinformatics*, 22(13):1658–1659, 2006. doi: 10.1093/bioinformatics/btl158.

Dmitry Meleshko, Iman Hajirasouliha, and Anton Korobeynikov. coronaSPAdes: from biosynthetic gene clusters to RNA viral assemblies. *Bioinformatics*, 2021. doi: 10.1093/bioinformatics/btab597.

Peter Menzel, Kim Lee Ng, and Anders Krogh. Fast and sensitive taxonomic classification for metagenomics with kaiju. *Nat. Commun.*, 7:11257, 2016. doi: 10.1038/ncomms11257.

Brian D Ondov, et al. Mash screen: high-throughput sequence containment estimation for genome discovery. *Genome Biol.*, 20(1):232, 2019. doi: 10.1186/s13059-019-1841-x.

Torbjørn Rognes, et al. VSEARCH: a versatile open source tool for metagenomics. *PeerJ*, 4:e2584, 2016. doi: 10.7717/peerj.2584.

Martin Steinegger and Johannes Söding. MMseqs2 enables sensitive protein sequence searching for the analysis of massive data sets. *Nat. Biotechnol.*, 35(11):1026–1028, 2017. doi: 10.1038/nbt.3988.

Md Vasimuddin, Sanchit Misra, Heng Li, and Srinivas Aluru. Efficient architecture-aware acceleration of BWA-MEM for multicore systems. In *2019 IEEE International Parallel and Distributed Processing Symposium (IPDPS)*, pages 314–324. IEEE, 2019. doi: 10.1109/IPDPS.2019.00041.

Derrick E Wood, Jennifer Lu, and Ben Langmead. Improved metagenomic analysis with kraken 2. *Genome Biol.*, 20(1):257, 2019. doi: 10.1186/s13059-019-1891-0.
