# Supplementary figures and images for: nf-core/viralmetagenome: A novel pipeline for untargeted viral genome reconstruction
Source: Bioinformatics. 2026 Apr 29;42(5):btag187. doi: 10.1093/bioinformatics/btag187 (PMC13141149; doi:10.1093/bioinformatics/btag187)

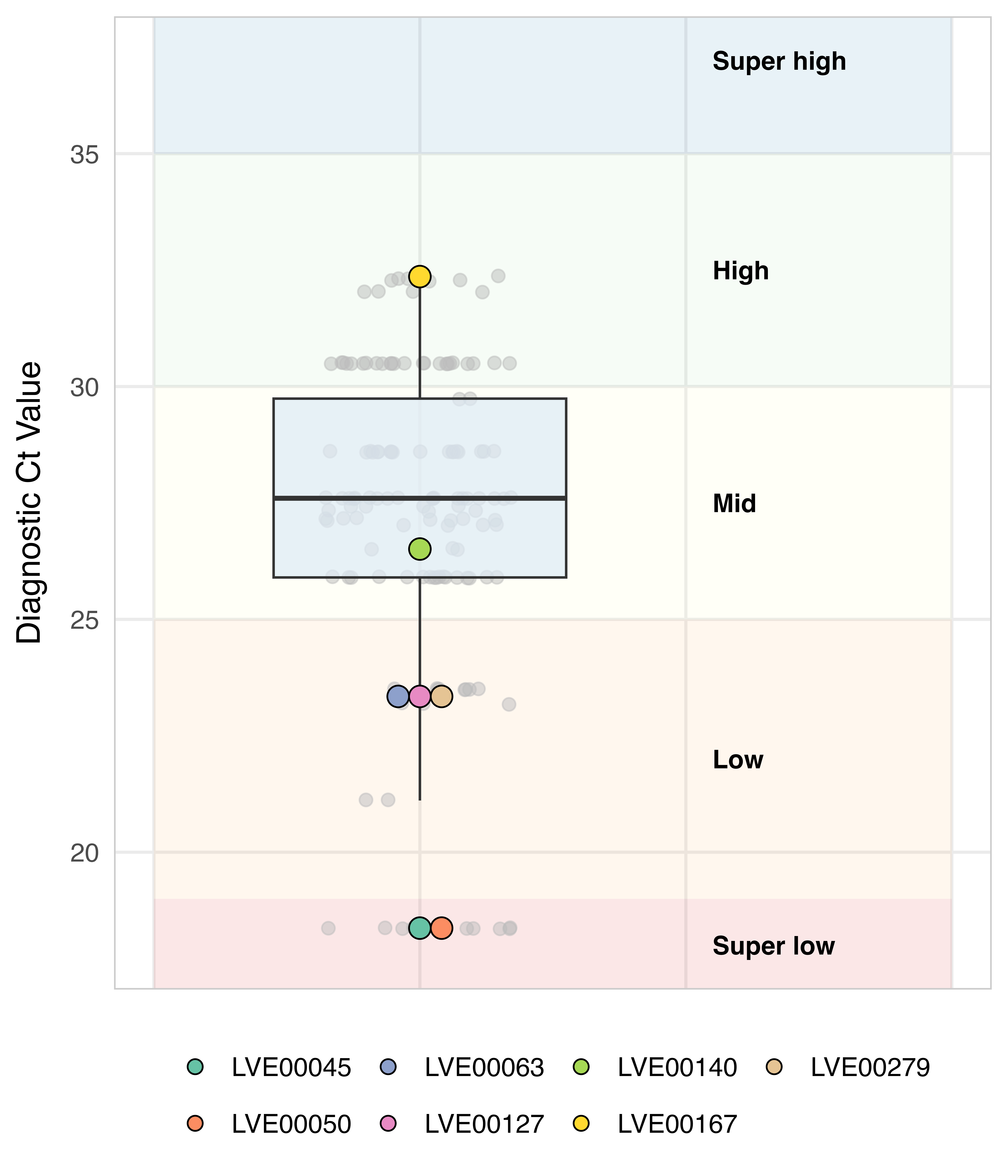

Supplement: btag187_Supplementary_Data [file btag187_supplementary_data.zip › supplfig2.png]
